# Development and Validation of the ATRAI Questionnaire to Assess Attitudes Toward Large Language Models in Clinical Setting (ATRAI-LLM)

**DOI:** 10.3390/ejihpe16070094

**Published:** 2026-06-30

**Authors:** Roman V. Reshetnikov, Yuriy A. Vasilev, Yuliya F. Shumskaya, Dina A. Akhmedzyanova, Yulya A. Alymova, Anton V. Vladzymyrskyy, Ilya A. Tyrov, Olga V. Omelyanskaya, Ivan A. Blokhin

**Affiliations:** 1 Research and Practical Clinical Center for Diagnostics and Telemedicine Technologies of the Moscow Health Care Department, 127051 Moscow, Russia; reshetnikovrv1@zdrav.mos.ru (R.V.R.); npcmr@zdrav.mos.ru (Y.A.V.); shumskayayf@zdrav.mos.ru (Y.F.S.); akhmedzyanovada@zdrav.mos.ru (D.A.A.); alymovaya@zdrav.mos.ru (Y.A.A.); vladzimirskijav@zdrav.mos.ru (A.V.V.); omelyanskayaov@zdrav.mos.ru (O.V.O.); 2 Moscow Health Care Department, 127006 Moscow, Russia; tyrovia@zdrav.mos.ru

**Keywords:** artificial intelligence, large language models, attitude, surveys and questionnaires

## Abstract

Background: Large language models (LLMs) are increasingly integrated into real-world medical practice as chatbots for answering clinical queries. However, the perceptions of this technology among its end-users remain understudied. Existing research on physicians’ attitudes toward LLMs relies on non-validated questionnaires, raising concerns about the accuracy and reliability of the findings. The aim of this study is to develop and validate a questionnaire to assess physicians’ attitudes toward LLM-based chatbots used as a reference tool for answering queries. Methods: The instrument was based on the previously developed and validated ATRAI-14 questionnaire assessing radiologists’ attitudes toward artificial intelligence. Items for the new questionnaire were formulated and refined through focus group testing. Validation involved 562 physicians of various specialties working in medical institutions within the Moscow healthcare system. Some respondents had prior experience working with medical LLMs. We assessed face, content, construct, and criterion validity. Criterion validity was evaluated through correlation between respondents’ self-assessed attitudes toward LLMs measured by visual analogue scale (VAS), and construct validity through confirmatory factor analysis. Results: The resulting ATRAI-LLM questionnaire comprised 19 items (8 in the background part and 11 in the main part). The questionnaire demonstrated acceptable internal consistency (Cronbach’s α = 0.770, McDonald’s ω_t_ = 0.830). It encompasses three domains: “Willingness to Use”, “Implementation Perspective”, and “Hopes and Fears.” Confirmatory factor analysis supported the three-factor structure, with satisfactory fit indices achieved (RMSEA = 0.05, CFI = 0.97, TLI = 0.96, SRMR = 0.03). Criterion validity was confirmed as acceptable with moderate correlation between the final score and VAS scores (Spearman’s rho 0.68, *p* < 0.001). Conclusions: ATRAI-LLM is a validated instrument for assessing physicians’ attitudes toward LLMs as a knowledge base.

## 1. Introduction

The widespread integration of artificial intelligence (AI) across all spheres of life creates a new technological reality accompanied by ambivalent public perceptions. These range from expectations of significant gains in efficiency and the automation of routine processes to concerns regarding potential risks associated with AI influence (Stein et al., 2024). Large language models (LLMs) are increasingly applied in healthcare (Meng et al., 2024) to automate the processing of medical data (Vasilev et al., 2025a), generate and summarize medical texts (Bednarczyk et al., 2025), and facilitate the education of patients (Unger et al., 2025) and physicians (Iqbal et al., 2025). However, these systems possess some limitations, including a tendency to generate convincing yet inaccurate information (“hallucinations”) and to amplify systemic biases present in training datasets (Athaluri et al., 2023). These shortcomings raise serious safety concerns regarding the integration of LLMs into clinical practice, where errors may have crucial consequences for patient health.

Retrieval-augmented generation (RAG) may improve the reliability of LLMs by grounding generated responses in external knowledge sources. This is particularly relevant when LLMs are used as clinical reference tools, where source traceability, up-to-date information, and hallucination reduction are important for physicians’ trust (Hang et al., 2025). Recent studies on RAG, including graph-based health-related fact-checking and contextual retrieval for rapidly evolving domain-specific knowledge (Conger et al., 2025), further highlight the need to evaluate clinicians’ attitudes toward grounded LLMs.

Despite the growing number of publications evaluating the capabilities of LLMs in solving specific medical tasks (Meng et al., 2024), existing studies are often focused on technical validation conducted by AI/ML computational science specialists. Such works rely on standardized statistical metrics (Barbella & Tortora, 2022; Reiter, 2018; Lavie & Agarwal, 2007) accompanied by expert evaluation, for which a range of tools have been developed (Vasilev et al., 2025b; Tam et al., 2024). The perception of this technology by its primary stakeholders—practicing clinicians—remains understudied. Physicians’ attitudes toward AI can influence both their expert assessment and the success of LLM implementation in real-world practice (Spotnitz et al., 2024). This is particularly relevant when LLM-based chatbots are used to answer questions, including those that require retrieving information from electronic health records (EHRs) (Reshetnikov et al., 2025).

Recent studies assessing physicians’, healthcare workers’, and students’ attitudes towards LLMs have relied on non-validated questionnaires (Vasilev et al., 2025b; Spotnitz et al., 2024; Reshetnikov et al., 2025). Despite this limitation, which the authors themselves acknowledge, the findings consistently indicate a positive overall perception of LLMs and a willingness to integrate them as assistive tools in clinical practice and education. Validated questionnaires for assessing physicians’ attitudes toward AI in general have been described in the literature (Stein et al., 2024), but they fail to capture the specific features of LLMs and their application in real-world clinical practice.

There is a methodological blind spot in existing research, necessitating the development of a validated questionnaire assessing physicians’ attitudes toward medical LLMs. Developing such a questionnaire would also help identify weaknesses in the implementation pipeline and support targeted improvements in educational programs to enhance physicians’ digital readiness as well as the development of specific administrative solutions.

Thus, the development of a reliable, valid questionnaire to assess physicians’ attitudes toward LLMs represents a necessary step for conducting methodologically sound research in this field. This is particularly important for healthcare initiatives integrating LLM-based chatbots into medical information systems (Reshetnikov et al., 2025). A reliable questionnaire would enable timely identification and management of various aspects of physicians’ attitudes toward LLMs.

We previously developed the ATRAI-14 questionnaire to assess radiologists’ attitudes toward artificial intelligence (AI) technologies (Vasilev et al., 2024). The instrument demonstrated acceptable internal consistency (Cronbach’s α = 0.78; 95% CI 0.68–0.83), high test–retest reliability (intraclass correlation coefficient [ICC] = 0.89; 95% CI 0.67–0.96; *p* < 0.05), and acceptable criterion validity (Spearman’s ρ = 0.73; *p* < 0.001).

Given these confirmed psychometric properties, we proceeded to develop an ATRAI-14-based questionnaire adapted for a different target population and expanded AI application scenarios. In this context, the acronym “ATRAI” is conceptualized as denoting a family of instruments designed to assess healthcare professionals’ attitudes toward AI-based digital technologies.

The aim of this study is to develop and assess the reliability and validity of a questionnaire designed to evaluate physicians’ attitudes toward LLM-based chatbots used as a tool for analyzing medical documents, including patient EHRs.

## 2. Materials and Methods

**Figure 1:**
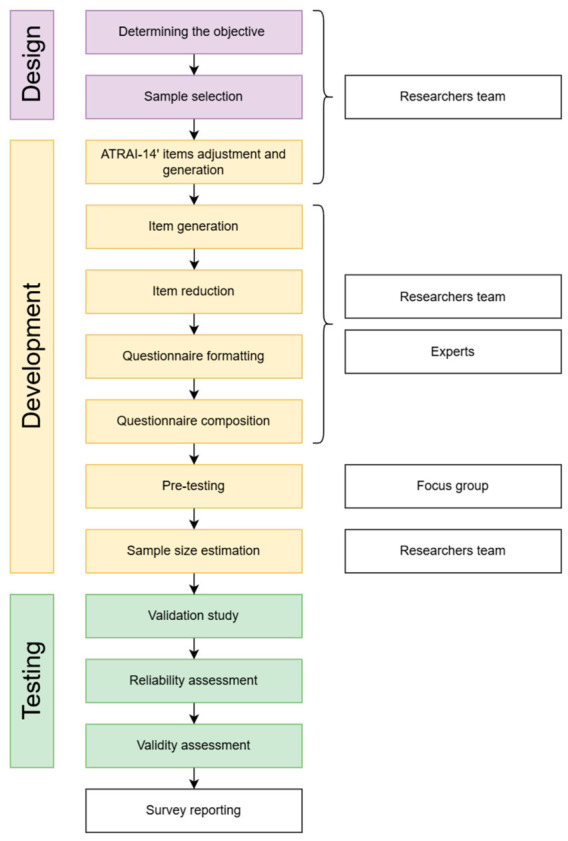
Study design.

The design of the questionnaire development and validation is presented in Figure 1.

### 2.1. Sample Selection

The questionnaire is intended for physicians of all specialties (outpatient and inpatient) and clinical directors.

### 2.2. Study Participants

The research team, which consisted of three physicians with at least three years of work experience, a sociologist, and three AI/ML computational scientists, was responsible for the questionnaire development.

Experts comprised two physicians with at least three years of work experience and two AI/ML computational scientists.

The focus group consisted of 15 physicians providing care in outpatient and inpatient settings. The population for reliability and validity assessment included 562 physicians of various specialties working in Moscow healthcare and taking part in the pilot project integrating an LLM-based chatbot into the Unified Medical Information and Analytical System (UMIAS) (Reshetnikov et al., 2025).

The survey was multicenter and included specialist physicians from 58 medical organizations: 5 medical organizations providing outpatient care to adults (including 22 satellite health centers), 5 medical organizations providing outpatient care to pediatric population (including 22 satellite health centers), and 4 multidisciplinary hospitals (3 providing care to adults and 1 to pediatric patients) within the Moscow Department of Health. Participating physicians represented a diverse range of specialties: general practitioners, pediatricians, infectious disease specialists, cardiologists, colorectal surgeons (proctologists), neurologists, nephrologists, otorhinolaryngologists, ophthalmologists, pulmonologists, rheumatologists, trauma and orthopedic surgeons, urologists, andrologists, general surgeons, endocrinologists, obstetricians, immunologists, gastroenterologists, and geriatricians. We also surveyed managerial staff, including chief physicians, branch heads, deputy chief physicians, and department heads.

The LLMs being implemented in healthcare were YandexGPT 5.1 Pro (Yandex LLC., Moscow, Russia) and GigaChat 2.0 (PJSC Sberbank of Russia, Moscow, Russia).

### 2.3. Questionnaire Development (Item Generation, Reduction, and Questionnaire Formatting)

We based the new instrument on the previously developed and validated ATRAI-14 questionnaire designed to assess radiologists’ attitudes toward artificial intelligence (Vasilev et al., 2024). It is important to note that Moscow Healthcare department radiologists are not only end-users of AI technologies but also participate in the development of specialized AI algorithms (Vasilev & Vladzymyrskyy, 2025). Physicians typically act only as end-users of fully developed software. Given the significant differences in how these two groups use AI, we had to adapt and refine several of the original questions.

We based the development of our questionnaire, as well as the parent ATRAI-14, on the theoretical domains framework, which is validated for use in implementation and behavior-change research. According to this framework, the behavior of healthcare workers toward an implemented innovation can be comprehensively assessed across 14 domains (Cane et al., 2012). Thus, we initially preserved the domain structure of the ATRAI-14 questionnaire. It contains a part related to the respondent’s demographics and professional background, followed by the main domains: “Trust”, “Implementation Perspective”, and “Hopes and Fears”.

In accordance with the theory of planned behavior (Ajzen, 1991), we define the attitude toward LLM assistant as a positive or negative intention of the healthcare professional to use the assistant in their clinical practice. This intention is based on the respondent’s beliefs on real-world capabilities of the LLM assistant, consequences of its implementation, and evaluation of those consequences.

The background part items were adapted to the new objectives and target population. We removed items related to radiology and experience with various imaging modalities and replaced them with items capturing more detailed information on respondents’ positions and specialties. We added an item to the background part to capture prior experience with LLMs in clinical practice. Furthermore, the questions assessing experience with LLMs in the UMIAS, originally in the “Familiarity” domain, were reduced to a single question and moved to the background part.

The “Trust” domain included five items designed to assess physicians’ trust in the LLM-based chatbot integrated into UMIAS. The “Implementation Perspective” domain comprised three items assessing anticipated adoption of the LLM assistant, while the “Hopes and Fears” domain contained three items identifying physicians’ concerns regarding its implementation. In total, the adapted questionnaire consisted of 19 items (8 in the background part and 11 in the main part).

The response formats included a five-point Likert scale, multiple-choice, and a five-point scale. For the Likert-based questions, response options ranged from 1 to 5, corresponding to extremely negative and extremely positive attitudes, respectively. Several items (T2, I1, I3) allowed multiple responses, and the total score for each of these items was calculated based on the number of selected options. To enhance the reliability of the collected data, we also included items with reversed scoring (T2) in the questionnaire. The use of reverse-scored items is a widely accepted methodological practice in survey design. Its primary purpose is to identify respondents who may be answering carelessly or exhibiting response bias, such as acquiescence (tendency to agree regardless of content).

Following the initial drafting of the questions, in-depth interviews with experts were conducted to evaluate the relevance and appropriateness of each item. Afterward, six items were revised.

The preliminary version of the questionnaire was then pilot-tested in a focus group. The focus group evaluated usability, clarity of the item wordings, and the appropriateness of the response options. Following this assessment, the research team determined whether revisions suggested by focus group members should be accepted. If similar comments were provided by the majority of physicians (10 or more) in the focus group, revisions were implemented without further discussion.

The ATRAI-LLM questionnaire was designed to assess physicians’ attitudes toward LLMs as practical tools rather than to evaluate their understanding of the underlying technical architecture. The survey items did not address technical details such as model architecture, training data, or algorithmic mechanisms, nor did they require respondents to possess such knowledge. From the perspective of the physician, the utility of an LLM assistant is judged by its performance in practice, independent of technical complexity. Within this framework, attitudes are shaped primarily by observed functionality: if the LLM performs poorly or produces errors, confidence in it is directly and negatively affected, regardless of the underlying technology.

### 2.4. Questionnaire Composition

We developed an electronic version of the questionnaire using the survey administration software “Yandex Forms”. The platform processes personal data only to the extent necessary for delivering the form and stores it in compliance with local data protection legislation. All data transmission between the respondent’s browser and Yandex’s servers is encrypted (HTTPS), and Yandex does not retain or publish the respondents’ answers beyond the period required for form fulfilment. No patient-identifying information (e.g., names, medical record numbers, or health-status details) was collected; the questionnaire asked only about physicians’ attitudes and practice-related experiences. Consequently, the data collection complied with relevant patient data protection requirements. Questions were presented in a series of linked pages (multiple-item screens) with accompanying electronic instructions.

Participants received a cover letter explaining the survey’s purpose. The first page of the electronic form presented the informed consent for participation in the study and for publication of the results, which participants needed to accept to proceed.

### 2.5. Pre-Testing

To evaluate how well respondents understood the questions and response options, four members of the research team conducted individual interviews with participants from the focus group who were similar to the sampling frame. The aim was to assess how the questions were interpreted and whether respondents’ understanding aligned with the original intent (Collins, 2003).

### 2.6. Sample Size Estimation

The minimum sample size for estimating a latent variable (attitude toward LLMs) based on three observed variables (“Trust”, “Implementation Perspective,” and “Hopes and Fears”) was 328 estimates (type I error rate 0.05, power 0.95) (Soper, 2026). For factor analysis with conditions of good agreement between sample and population, a wide level of communality, and three factors with at least three variables per factor, the estimation of minimum necessary sample size was 450 participants (Mundfrom et al., 2005).

### 2.7. Reliability and Validity Assessment

A validation study was conducted to evaluate the reliability and validity of the questionnaire. Participants were provided access to the electronic version of the ATRAI-LLM. Following data collection, reliability and validity analyses were performed; the statistical methods applied are summarized in Table 1.

Reliability was assessed based on internal consistency.

Four types of validity were evaluated: face, content, construct, and criterion validity. Face and content validity were assessed by experts (*n* = 4). The following questions were considered: “Does the questionnaire measure the intended construct?” and “Does the questionnaire adequately cover all key aspects of the domain?” Each expert and research team member provided a binary response (“yes” or “no”) for each item. An item was considered acceptable if 75% of experts (≥3) provided an affirmative response.

Construct validity was examined using confirmatory factor analysis (CFA) to test the hypothesis that the observed data fit the proposed domain structure and to identify items requiring modification or removal. Criterion validity was assessed by comparing questionnaire scores with respondents’ self-reported attitudes measured on a visual analogue scale (VAS) ranging from 0 to 10, where 0 indicated the most negative attitude and 10 the most positive attitude.

### 2.8. Statistical Data Analysis

Data were processed using R version 4.3.1 with the psych (2.4.6), lavaan (0.6-18), and ltm (1.2-0) packages. Calculated values were interpreted according to Table 1 with assessment of statistical significance. A *p*-value < 0.05 was considered statistically significant for all tests.

## 3. Results

### 3.1. Questionnaire Development

The questionnaire assessing physicians’ attitudes toward LLMs used in their practice (ATRAI-LLM) consists of two parts: a background part and a main part, the latter contributing to the total score (Appendix A). The structure of the questionnaire is presented in Table 2. Scores for the main part items were summed for each respondent to generate the final ATRAI-LLM score.

### 3.2. Testing

A total of 714 respondents completed the ATRAI-LLM questionnaire. After initial data cleaning based on specialty (retaining only representatives of the target population: physicians and clinical directors), 562 respondents were included in the analysis.

Notably, despite having access to LLMs within UMIAS, 159 respondents (28.3%) reported not using LLMs in their clinical practice, 217 (38.6%) reported occasional use, and 186 (33.1%) reported regular use of LLMs in their work.

**Figure 2:**
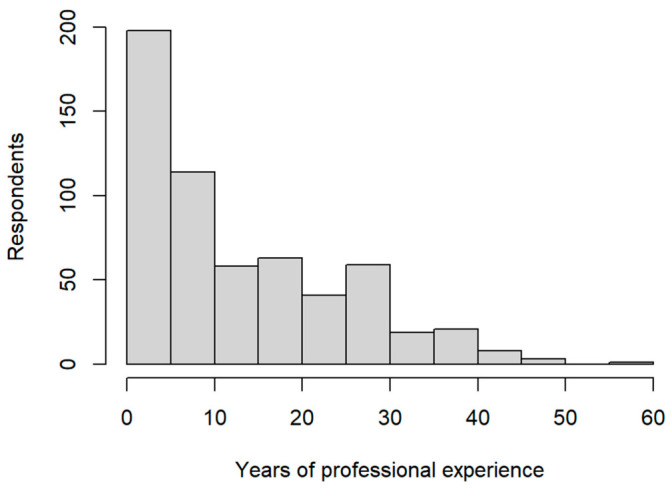
Distribution of respondents by years of professional experience.

Most respondents (54%) had less than 10 years of professional experience (Figure 2).

**Figure 3:**
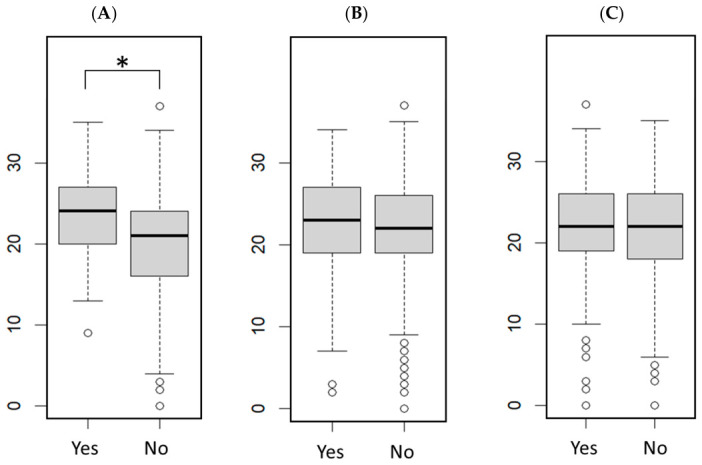
Boxplots of ATRAI-LLM scores in accordance with the demographic characteristics. (**A**) Subgroups of respondents using (“Yes”) and not using (“No”) LLMs in clinical practice. (**B**) Subgroups of respondents participating (“Yes”) and not participating (“No”) in research activities. (**C**) Subgroups of respondents working (“Yes”) and not working (“No”) with pediatric patients. Statistically significant differences are marked with (*).

A subgroup analysis was conducted based on demographic variables obtained from the survey. Respondents who used LLMs in their clinical practice demonstrated a more optimistic attitude toward these technologies (*p*-value < 0.001). The difference between the groups corresponded to the medium effect size (rank-biserial correlation r = 0.370, 95% CI: [0.290; 0.450]). No significant differences were observed between physicians working with pediatric versus adult populations (*p*-value = 0.245), nor between those with and without research experience (*p*-value = 0.235) (Figure 3).

### 3.3. Validation Results

#### 3.3.1. Face and Content Validity

According to the assessment of the research team and expert group, all items in the final version of the ATRAI-LLM were considered valid (Table 3).

#### 3.3.2. Internal Consistency

**Figure 4:**
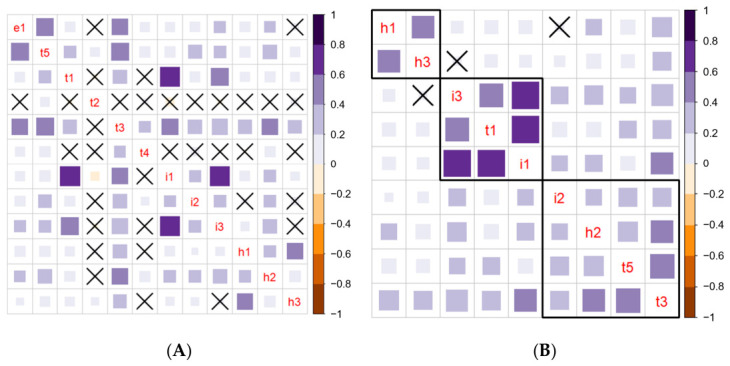
Correlation matrices of questionnaire items. Crosses (✕) below the matrix in a diagonal direction indicate statistically insignificant correlations. Above the diagonal, crosses indicate correlations that did not remain significant after Holm correction for multiple comparisons. (**A**) Correlation matrix for all items. (**B**) Correlation matrix for items grouped into domains based on hierarchical clustering; domains (“Willingness to Use,” “Implementation Perspective,” “Hopes and Fears”) are marked by squares from bottom to top.

The correlation matrix for the main part of ATRAI-LLM demonstrated a significant negative correlation between items T2 and I1 (Figure 4A). No other significant negative correlations were identified among the items contributing to the overall attitude score (T1–H3), indicating that the items do not conflict in the directional assessment of attitudes toward LLMs. It is important to note that the questions T3 (“Would you like LLMs for physicians to be widely implemented and actively used in clinical practice?”) and I3 (“Which components of your daily professional activity do you expect to change with the use of LLM in the next 1–2 years?”) stand out as the most correlated with the other parts of the questionnaire. Items T2 and T4 also stood out due to the near absence of significant correlations with other questionnaire items or with each other.

Although items T2 and T4 were excluded from the total score, they were retained in the questionnaire because they provide important complementary information. Item T2 reflects physicians’ reliance on their own knowledge versus external sources of expertise. The question is essential for identifying the need for educational interventions addressing the lack of domain knowledge, risks of LLM hallucinations, and inappropriate reliance on AI-generated content. Item T4 captures preferred modes of interaction with LLM systems, providing insight into acceptable implementation pathways and supporting managerial decisions regarding optimal integration of LLM functionality into clinical workflows.

Based on the correlation analysis results, the final structure of the questionnaire was established, consisting of 19 items (8 background and 11 main). The scale of domain structure changes in comparison with the parent ATRAI-14 questionnaire necessitated reinterpreting the domains.

Domain 1 had three items: T1 (most trustworthy topics), I1 (most-used functions), and I3 (LLM-associated changes in professional practice). The three questions related to perspectives of LLM integration into the current healthcare system; therefore, we labeled this domain as “Implementation Perspective”.

The questions that loaded highly on domain 2 were T3 (opinion on wide LLM implementation in clinical practice), T5 (trustworthiness of LLM-retrieved information from EHR), I2 (opinion on who should pay for LLM assistant), and H2 (LLM-associated changes in physicians’ workload). All these items relate to entities that encourage or discourage the use of an LLM assistant. Therefore, we labeled this domain as “Willingness to Use”.

Domain 3 had only two items, H1 (expectations for LLM-associated changes in status) and H3 (expectations for LLM-associated changes in salary). These items reflect physicians’ concerns regarding their professional future following the implementation of LLMs, and we labeled the domain as “Hopes and Fears”.

Internal consistency was assessed by Cronbach’s alpha calculation for the combined domain section of the questionnaire reflecting attitudes toward LLMs. Cronbach’s alpha was 0.770 (95% CI [0.731, 0.800]), indicating satisfactory internal consistency of the instrument. Based on McDonald’s omega, overall internal consistency was ω_t_ = 0.830 and hierarchical ω_h_ = 0.610, indicating good internal consistency of the instrument.

#### 3.3.3. Construct Validity

Construct validity was evaluated using confirmatory factor analysis. The three-factor model demonstrated satisfactory fit indices: RMSEA = 0.05, CFI = 0.97, TLI = 0.96, SRMR = 0.03. A single-factor model was tested as a comparator and showed poor fit indices (RMSEA = 0.15, CFI = 0.74, TLI = 0.65, SRMR = 0.09). Factor loadings for the three-factor model are presented in Table 4.

Factor loadings exceeded 0.5 for 7 of the 9 items, indicating good correspondence between the data and the proposed model. For items I2 and H3, the loadings were 0.31 and 0.47, respectively—lower than for other items but still indicative of a meaningful association with the corresponding factor (domain). Thus, the factor analysis supported the domain structure of the instrument.

**Figure 5:**
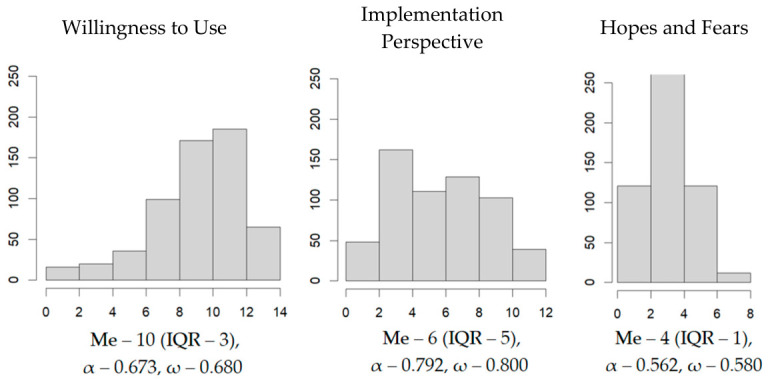
Distribution of ATRAI-LLM domain scores. *X*-axis: score, *Y*-axis: number of respondents.

Instructions for interpreting item-level responses are provided in Appendix B. The ATRAI-LLM score is calculated as the sum of points from all scored items (T1, T3, T5, I1, I2, I3, H1, H2, and H3). The maximum possible total score is 36, and the minimum ATRAI-LLM score is 0. For individual domains, the “Willingness to Use” score ranges from 0 to 16, the “Implementation Perspective” score ranges from 0 to 12, and the “Hopes and Fears” score ranges from 0 to 8 (Figure 5). An example of questionnaire completion with total score calculation is presented in Appendix C.

#### 3.3.4. Criterion Validity

**Figure 6:**
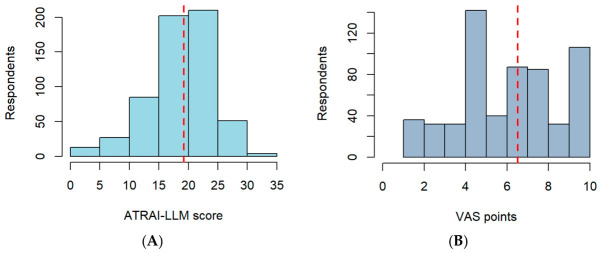
Distributions of ATRAI-LLM scores (**A**) and VAS points (**B**). Red dotted lines indicate mean values.

In the analyzed sample (562 respondents), the minimum observed ATRAI-LLM score was 0 (indicating an extremely negative attitude), and the maximum was 33 (out of a possible 36, indicating a strongly positive attitude). The median score was 20 points (IQR 16 to 23 points). The distribution of scores is presented in Figure 6A. The median self-assessment according to the VAS was 7 points (IQR 5 to 8 points) (Figure 6B).

**Figure 7:**
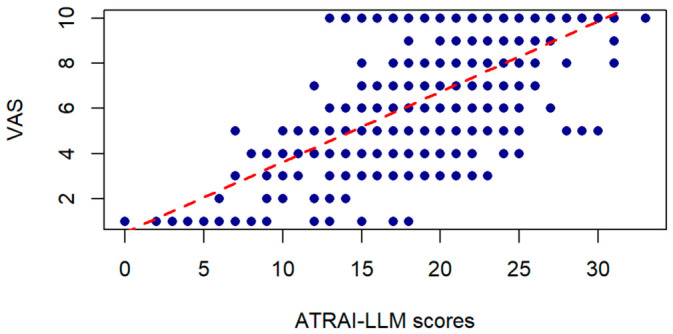
Scatterplot of ATRAI-LLM scores and self-assessed attitudes toward LLMs on the visual analogue scale. The red dotted line indicates the trend.

The questionnaire demonstrated acceptable criterion validity with moderate correlation between the final score and respondents’ self-assessed attitudes toward LLMs on the visual analogue scale (Figure 7) (Spearman’s rho 0.68, *p* < 0.001). This finding confirms that the developed instrument measures attitudes toward LLMs.

We have also estimated the predictive validity of the ATRAI-LLM questionnaire by a correlational analysis of relationships between the F1 item score (“Do you use the LLM assistant in your clinical practice?”) with the total ATRAI-LLM score and the individual domains’ scores. The real-world behavior was positively related to the ATRAI-LLM total score, with a Spearman correlation coefficient of 0.32 (*p* < 0.001). Among the individual domains, the “Willingness to Use” domain had the strongest correlation with the F1 item score (Spearman’s rho 0.40, *p* < 0.001).

## 4. Discussion

We developed and validated the ATRAI-LLM questionnaire to assess physicians’ attitudes toward LLMs in healthcare specifically when used as a tool for answering clinical queries. The final questionnaire comprised 19 items: 8 in the background part and 11 in the main part, 9 of which contributed to scoring. The validation results confirmed the three-domain structure: the three-factor model demonstrated satisfactory fit indices (RMSEA = 0.05, CFI = 0.97, TLI = 0.96, SRMR = 0.03). Three domains were retained: “Willingness to Use,” “Implementation Perspective,” and “Hopes and Fears”. Criterion validity demonstrated statistically significant yet moderate correlation between the ATRAI-LLM score and visual analogue scale assessment (Spearman’s rho = 0.68, *p* < 0.001). The correlation coefficient observed in the present study was close to the ATRAI-14 study (Vasilev et al., 2024).

The instrument demonstrated acceptable internal consistency (Cronbach’s alpha 0.770, 95% CI [0.731, 0.800], McDonald’s omega ω_t_ = 0.830, ω_h_ = 0.610). This result exceeds the commonly accepted threshold of 0.7 for research instruments, supporting its use both in scientific studies and in practical healthcare settings, given that end-user attitudes directly influence the success of technological implementation (Spotnitz et al., 2024). Moreover, no ceiling or floor effects were observed, as scores were well-distributed across the entire range (median—20 points, maximum—33 out of 36) with approximately normal distribution (Figure 6A). Additionally, the absence of a maximum score indicates that current implementation of LLMs is not sufficient to gain the full trust of physicians.

ATRAI-LLM comprises three domains that assess different aspects of attitudes toward LLMs. The “Willingness to Use” domain measures the respondent’s perception of LLM assistant quality. The “Implementation Perspective” domain reflects respondents’ awareness of LLM assistant usefulness. The “Hopes and Fears” domain captures perceptions of how LLMs may influence physicians’ careers in terms of salary and professional prestige. We defined the attitude toward an LLM assistant as a positive or negative intention of the healthcare professional to use the assistant in their clinical practice. According to Conner (Conner, 2001), there are three variables determining the intention: (1) the respondent’s evaluation of their behavior, (2) subjective norms, reflecting the respondent’s beliefs on how their peers and significant others would perceive the respondent’s behavior, and (3) the degree of control the respondent has over their behavior in the current situation. Therefore, the construct definition of attitude toward LLMs implemented in the ATRAI-LLM questionnaire fits well with the concept of intention-to-use determinants, having a correspondence between the “Willingness to Use” domain and behavior evaluation, the “Implementation Perspectives” domain and the degree of control over the situation, and the “Hopes and Fears” domain and subjective norms.

This domain-based questionnaire structure enables not only the assessment of physicians’ overall attitudes toward LLM assistants but also the identification of weaker domains. ATRAI-LLM may help identify specific barriers and problem areas in the integration of LLMs into real-world clinical practice, including hospital policy, thereby informing the development of targeted organizational and educational interventions. In particular, domain-specific results may support the design of training programs aimed at improving physicians’ competencies in the responsible use of LLM-generated information. Furthermore, repeated administration of the instrument during different stages of system deployment may facilitate longitudinal monitoring of changes in physicians’ attitudes. More specifically, repeated administration before deployment, after training, and after several months of use could help monitor whether physicians’ concerns decrease, whether trust increases appropriately, and whether overreliance risks emerge. This structured feedback would be essential to optimize user acceptance and ensure sustainable integration of LLM-based tools into clinical workflows.

This is also relevant for retrieval-supported LLM systems, where physicians’ attitudes may depend not only on the perceived usefulness of the generated response but also on the transparency of retrieved sources, the perceived reliability of grounding information, and the integration of these functions into clinical workflows. In this context, ATRAI-LLM may help identify whether barriers to adoption are related to general unwillingness to use LLMs, concerns about implementation, or specific doubts regarding source-grounded clinical information.

Physicians’ attitudes toward LLMs should also be interpreted as task-dependent. Previous studies suggest that clinicians may perceive LLMs more favorably when they are used for low-risk administrative or informational tasks, such as documentation support, summarization, or generation of patient educational materials, whereas greater caution is expressed when LLMs are expected to support diagnosis, treatment planning, or clinical judgment (Blease et al., 2025; Tangadulrat et al., 2023). In a qualitative study of UK general practitioners, Blease et al. showed that physicians recognized the potential of LLMs for documentation-related tasks but raised concerns about clinical judgment, accountability, and operational uncertainty. Similarly, Tangadulrat et al. reported that physicians were more cautious than medical students regarding the use of ChatGPT for treatment guidance and medical education, while both groups viewed its use for patient educational materials more positively. These findings are consistent with the structure of ATRAI-LLM, which separates willingness to use, implementation perspectives, and hopes and fears, and may help identify whether physicians’ concerns are related to specific high-risk clinical applications rather than to LLMs in general.

In comparison with the ATRAI-14 questionnaire, the ATRAI-LLM “Hopes and Fears” domain was reduced to two items. While some concerns can be expressed about content validity, sensitivity, and reliability of two-item factors, there is evidence available that even single-item scales can serve as substitutes for 20-item measures of health-related parameters (Cunny & Perri, 1991). Moreover, shorter surveys were shown to be reliable while producing higher response and completion rates (Kost & Correa da Rosa, 2018). Currently, we are performing a study to test this observation in relation to the ATRAI-LLM questionnaire.

According to the results of the factor analysis, items I2 and T1 demonstrated a redistribution of factor loadings and were assigned to factors other than those hypothesized a priori during the scale development process. The observed differences in the factor structure may be attributable to variations in the target population of the instrument and the contextual setting in which AI technologies are applied. In the present study, substantial heterogeneity was observed among respondents, as the sample comprised physicians from diverse clinical specialties. In contrast, the ATRAI-14 study involved only radiologists. This distinction is crucial, as AI has been integrated into radiology for an extended period of time; many algorithms have become commonplace in clinical practice and demonstrate high levels of accuracy. Conversely, LLMs often exhibit errors (Athaluri et al., 2023), the detection of which can significantly depend on the physician’s level of expertise.

Despite existing attempts to evaluate physicians’ attitudes toward LLMs, a major methodological limitation of previous studies is the use of non-validated questionnaires (Spotnitz et al., 2024). In a study by Xu et al. (2024), the questionnaire included demographics, AI baseline proficiency and usage, perception of LLMs, and implications of AI in medical education and healthcare. However, the absence of confirmatory factor analysis prevented definitive conclusions about whether the instrument truly measured the constructs it was intended to measure. Moreover, the authors did not perform an a priori sample size calculation. Their final sample included 102 medical students rather than practicing physicians, which restricts the applicability of the findings to real-world clinical practice.

In Sumner et al. (2025), the sample size was larger (1083 respondents), but the population was highly heterogeneous, including practicing physicians, nurses, hospital administrative staff, and medical students. Furthermore, the questionnaire domains were, in our view, not designed to evaluate the respondent’s personal stance on the potential implementation and use of LLMs in clinical practice. The use of convenience sampling also limits the representativeness of the data and the generalizability of the findings to the broader physician population.

Spotnitz et al. (2024) assessed physicians’ attitudes toward LLMs and their comfort level in using them for various clinical, educational, and research tasks. Participants expressed favorable attitudes toward most evaluated AI-assisted tasks: nearly 70% (16 out of 23) received positive ratings from at least half of the respondents, with the greatest support observed for applications involving data analysis, modeling outbreaks, creating training cases, and clinical decision support. In contrast, tasks involving direct patient communication or complex content generation—such as responding to patient questions about radiology reports or writing original scientific manuscripts—received the fewest positive and the most negative ratings. Thus, the questionnaire used in that study can be viewed primarily as a tool for assessing the acceptability of using LLMs for different tasks. Recruitment was conducted through convenience sampling, and the sample consisted of 30 physicians from a single medical center, limiting the generalizability of the findings. Moreover, although the authors state that they used a valid instrument, standard validation procedures were not performed.

Our questionnaire enables the assessment of physicians’ attitudes toward LLMs used in medicine as tools for answering medical questions. A key advantage of the instrument is its comprehensive validation, confirming its robustness across four criteria of validity—face, content, construct, and criterion, as well as its reliability. This supports the high quality and interpretability of the data obtained with this tool. Importantly, the potential value of ATRAI-LLM extends beyond quantitative assessment; it also provides rich material for qualitative analysis. In our opinion, it is best to pair the ATRAI-LLM survey results with the data on actual LLM usage. There is a gap between self-reported attitude and real-life behavior (Vasilev et al., 2024), and the modern view on the problem dictates integration of additional sources of data to reflect the respondents’ experiences in real-world settings (Shankar et al., 2025).

This study has several limitations. During the development and testing stages, we surveyed only physicians from the Moscow Healthcare Department and validated the questionnaire exclusively within a Russian-speaking population. Furthermore, a substantial proportion of participants (71.7%) reported prior use of LLM-based chatbots within the UMIAS, which may represent a potential confounding factor in the analysis, as the quality of the LLMs deployed could have influenced respondents’ attitudes. The quality and performance of this specific LLM implementation may have influenced the respondents’ overall perception of LLMs. We did not conduct test–retest reliability analysis due to the inherently dynamic nature of LLMs as a rapidly evolving system. Respondents’ attitudes, experiences, and access to LLMs can change significantly even within two weeks, making traditional test–retest procedures potentially inappropriate, as any observed instability could reflect genuine change rather than measurement error. The concept of the test–retest analysis relies on the assumptions of perfectly stable true scores, which in our case is clearly violated, thus introducing bias into the analysis. Simulated data of Groh show that decreasing true score stability indeed biases test–retest metric estimates (Groh, 2026). Therefore, ATRAI-LLM questionnaire provides the attitude estimate at the time of the survey. Nevertheless, to partially address concerns regarding the internal coherence of our measures, we conducted an alternative reliability check by analyzing the relationship between a behavioral item assessing actual LLM usage (F1) and overall attitudes toward LLMs. Additionally, the ATRAI-LLM questionnaire was adapted from the previously validated ATRAI-14 instrument, which may have influenced its final structure and item count. We mitigated this potential bias via validation of the new questionnaire, confirming its reliability and validity. Finally, the age and gender of respondents were not collected to preserve anonymity. Future studies should include broader demographic variables to assess possible differences in attitudes and usage patterns.

In subsequent publications, we plan to report analyses of physicians’ attitudes toward LLMs and the factors influencing these attitudes.

## 5. Conclusions

In this study, we developed and validated a specialized questionnaire to assess physicians’ attitudes toward the use of LLM-based chatbots as tools for answering medical questions. The instrument comprises three key domains: “Willingness to Use,” “Implementation Perspective,” and “Hopes and Fears.” It includes 19 items (8 background and 11 main). It demonstrated satisfactory reliability and validity. Thus, the ATRAI-LLM questionnaire represents a robust tool that not only enables quantitative assessment of overall attitudes toward LLM use in the specified context but also supports qualitative analysis by identifying specific barriers and areas of concern.

## Figures and Tables

**Table 1:** Methods used to assess reliability and validity.

Dimension	Method	Thresholds
Internal consistency	Cronbach’s alpha McDonald’s omega (ω)	≤0.5—unacceptable >0.5—poor >0.6—questionable >0.7—acceptable >0.8—good >0.9—excellent (Dunn et al., 2014)
Face validity	Experts evaluate whether the questionnaire measures what it intends to measure.	More than 75% of experts (Diamond et al., 2014)
Content validity	Experts evaluate whether questionnaire content accurately assesses all fundamental aspects of the topic	More than 75% of experts (Diamond et al., 2014)
Construct validity	Confirmatory factor analysis	Comparative Fit Index (CFI) ≥ 0.9 Root Mean Square Error of Approximation (RMSEA) < 0.08 Standardized Root Mean Squared Residual (SRMR) < 0.08 Tucker Lewis Index (TLI) ≥ 0.9 (Brown, 2015)
Criterion validity	Correlation with visual analogue scale	<0.10—negligible correlation 0.10–0.39—weak correlation 0.40–0.69—moderate correlation 0.70–0.89—strong correlation ≥0.90—very strong correlation (Schober et al., 2021)

**Table 2:** Questionnaire structure.

№	Item	Item Code
Demographic and background part		
1	State your job title	P1
2	Specialty	P2
3	State the type of medical facility you work at	P3
4	What is the age of the patients you work with?	P4
5	State your total years of professional experience (excluding residency)	P5
6	Do you take part in research related to your medical specialty?	P6
7	Do you use large language models (Gemini, ChatGPT, DeepSeek, etc.) in your clinical practice?	P7
8	Do you use the question-answering LLM-based chatbot integrated into Health information system (hereinafter, LLM) in your clinical practice?	F1
Main part		
9	For which of the following topics would you trust the informational output from LLM?	T1
10	If LLM’s response raises doubts, would you cross-check the information?	T2
11	Would you like LLMs for physicians to be widely implemented and actively used in clinical practice?	T3
12	Which way of interacting with LLM would be preferable for you?	T4
13	Do you trust the information provided by LLM when it retrieves data from the patient’s EHR?	T5
14	In your opinion, which of the listed functions of LLM would be most useful and widely used in physicians’ work?	I1
15	In your opinion, who should pay for access to LLM in healthcare?	I2
16	Which components of your daily professional activities do you expect to change with the use of LLMs in the next 1–2 years?	I3
17	In your opinion, will the widespread use of LLMs affect the prestige of the profession in the next five years?	H1
18	In your opinion, will the widespread use of LLMs affect physicians’ workload?	H2
19	In your opinion, will the widespread use of LLMs affect physicians’ salary over the next five years?	H3

**Table 3:** Face and content validity of the questions.

№	Item Code	Face Validity Experts, *n* (%)	Content Validity Experts, *n* (%)
**1**	P1	4 (100)	4 (100)
**2**	P2	4 (100)	4 (100)
**3**	P3	4 (100)	4 (100)
**4**	P4	4 (100)	4 (100)
**5**	P5	4 (100)	4 (100)
**6**	P6	4 (100)	4 (100)
**7**	P7	4 (100)	4 (100)
**8**	F1	4 (100)	4 (100)
**9**	T1	3 (75)	3 (75)
**10**	T2	3 (75)	4 (100)
**11**	T3	4 (100)	4 (100)
**12**	T4	4 (100)	3 (75)
**13**	T5	4 (100)	4 (100)
**14**	I1	3 (75)	4 (100)
**15**	I2	4 (100)	4 (100)
**16**	I3	3 (75)	4 (100)
**17**	H1	4 (100)	3 (75)
**18**	H2	4 (100)	4 (100)
**19**	H3	3 (75)	3 (75)

**Table 4:** Factor loadings for three-factor model.

Item	Standardized Factor Loadings (SE)			*p*-Value
	F1	F2	F3	
T1	0.91 (0.05)	-	-	<0.001
T3	-	0.86 (0.04)	-	<0.001
T5	-	0.56 (0.04)	-	<0.001
I1	0.97 (0.04)	-	-	<0.001
I2	-	0.31 (0.03)	-	<0.001
I3	0.73 (0.04)	-	-	<0.001
H1	-	-	0.66 (0.06)	<0.001
H2	-	0.53 (0.04)	-	<0.001
H3	-	-	0.47 (0.04)	<0.001

## Data Availability

The original contributions presented in the study are included in the article; further inquiries can be directed to the corresponding author.

## Abbreviations

The following abbreviations are used in this manuscript:

AI	Artificial Intelligence
LLM	Large Language Model
IQR	Interquartile Range
UMIAS	Unified Medical Information and Analytical System of Moscow
EHR	Electronic Health Record
RMSEA	Root Mean Square Error of Approximation
SRMR	Standardized Root Mean Squared Residual
TLI	Tucker Lewis Index
CFI	Comparative Fit Index
ATRAI	Attitude of Radiologists Toward Radiology AI

## Appendix A. Questionnaire to Assess Attitudes Toward Large Language Models in Clinical Setting (ATRAI-LLM)

1. P1 State your job title

○	Physician (clinical specialist)
○	Head of department
○	Other

If “Other,” please specify

__________________________

2. P2 Specialty

__________________________

3. P3 State the type of medical facility you work at

○	Outpatient department
○	In-patient department
○	Day-case unit

4. P4 What is the age of the patients you work with?

○	<18 years old
○	≥18 years old
○	I work with patients of all ages

5. P5 State your total years of professional experience (excluding residency)

○	Less than 1 year
○	More than 1 year

If “More than 1 year,” specify the number of years

__________________________

6. P6 Do you take part in research related to your medical specialty?

○	Yes
○	No

7. P7 Do you use large language models (Gemini, ChatGPT, DeepSeek, etc.) in your clinical practice?

○	Yes
○	No
○	No, but I would like to

8. F1 Do you use the question-answering LLM-based chatbot integrated into Health information system (hereinafter, LLM) in your clinical practice? (one answer)

	Answer
○	Yes, regularly, for various tasks (e.g., searching for drug information, interpreting test results, etc.)
○	Yes, regularly, but only for a specific clinical task (e.g., only for test–result interpretation or only for drug-information search)
○	Yes, but irregularly
○	Not yet, but I plan to
○	No, and I do not plan to

9. T1 For which of the following topics would you trust the informational output from LLM?

◻	Regulatory documents, health information system functions, templates, sick-leave documentation
◻	Medications (INN, dosage forms, packaging, indications, dosing regimens, adverse reactions, contraindications, precautions, overdose, drug interactions)
◻	ICD-10 codes, diagnosis formulation, classifications, scoring systems, calculations
◻	Etiology, pathogenesis, epidemiology, clinical presentation, treatment of diseases
◻	General medical advice (diet, lifestyle, physical activity, etc.), vaccination, preparation for diagnostic tests
◻	Parameters, reference ranges, interpretation of diagnostic methods (physical examination, laboratory, and imaging data)
◻	I would not trust information on any topic

10. T2 If LLM’s response raises doubts, would you cross-check the information? (one answer)

	Answer
○	Yes
○	Mostly yes
○	Difficult to answer
○	Mostly no
○	No

11. T3 Would you like LLMs for physicians to be widely implemented and actively used in clinical practice? (one answer)

Label		Answer
4	○	Yes
3	○	Mostly yes
2	○	Difficult to answer
1	○	Mostly no
0	○	No

12. T4 Which way of interacting with LLM would be preferable for you? (one answer)

	Answer
○	LLM provides both EHR-derived and reference information
○	LLM provides only reference information
○	LLM provides only EHR-derived information
○	LLM provides EHR-derived and/or reference information with mandatory of source citations
○	I would prefer not to use an LLM within the health information system (e.g., UMIAS)

13. T5 Do you trust the information provided by the LLM when it retrieves data from the patient’s EHR? (one answer)

Label	○	Answer
4	○	Yes
3	○	Mostly yes
2	○	Difficult to answer
1	○	Mostly no
0	○	No

14. I1 In your opinion, which of the listed functions of LLM would be most useful and widely used in physicians’ work? (select all that applies)

◻	Summarizing the patient’s EHR for rapid review
◻	Providing reference information on regulatory documents, health information system functionality, templates, and sick-leave documentation
◻	Providing drug-related reference information
◻	Providing reference information on ICD-10 codes, diagnosis formulation, classifications, scoring systems, and calculations
◻	Providing reference information on parameters, reference ranges, and interpretation of diagnostic methods
◻	Providing reference information on etiology, pathogenesis, epidemiology, clinical presentation, and treatment of diseases
◻	Providing general medical advice (diet, lifestyle, physical activity, etc.), vaccination, and preparation for diagnostic tests
◻	Pre-populating medical documentation
◻	Assisting with communication with colleagues and patients
◻	I do not consider LLM useful
◻	Other (specify)

15. I2 In your opinion, who should pay for access to LLM in healthcare? (one answer)

Label		Answer
2	○	Public healthcare system (compulsory health insurance)
2	○	Patient’s insurance provider (voluntary health insurance)
3	○	The medical organization implementing LLM
4	○	The physician using LLM
1	○	LLM developer company
0	○	I do not anticipate widespread use of LLMs
2	○	Other (specify)

16. I3 Which components of your daily professional activities do you expect to change with the use of LLMs in the next 1–2 years? (select all that applies)

◻	Communication with patients
◻	Communication with colleagues
◻	Searching medical information resources
◻	Completing medical documentation
◻	Clinical decision-making (patient management)
◻	Technical aspects of working with the health information system
◻	Interpretation of diagnostic results
◻	Analysis of patients’ EHRs
◻	I do not think LLMs will have an impact
◻	Other (specify)

17. H1 In your opinion, will the widespread use of LLMs affect the prestige of the profession in the next five years? (one answer)

Label		Answer
2	○	Will not affect/difficult to answer
1	○	Perhaps, the prestige will decrease slightly
0	○	Yes, the prestige will decrease substantially
3	○	Perhaps, the prestige will increase slightly
4	○	Yes, the prestige will increase substantially

18. H2 In your opinion, will the widespread use of LLMs affect physicians’ workload? (one answer)

Label		Answer
4	○	The workload will decrease substantially
3	○	The workload will decrease somewhat
2	○	Will not affect/difficult to answer
1	○	The workload will increase somewhat
0	○	The workload will increase substantially

19. H3 In your opinion, will the widespread use of LLMs affect physicians’ salary over the next five years? (one answer)

Label		Answer
2	○	Will not affect/difficult to answer
1	○	The salary will decrease somewhat
0	○	The salary will decrease substantially
3	○	The salary will increase somewhat
4	○	The salary will increase substantially

## Appendix B. ATRAI-LLM Scoring

For items T3, T5, I2, H1, H2, and H3, the score corresponds to the number of the selected answer (listed under “label”).

For multiple-choice items, the following scoring system is applied:

Item T1:

0 points: “I would not trust information on any topic”;

1 point: 1 option selected;

2 points: 2 options selected;

3 points: 3–4 options selected;

4 points: 5–6 options selected;

Item I1:

0 points: “I do not consider LLM useful”;

1 point: 1–2 options selected;

2 points: 3–4 options selected;

3 points: 5–7 options selected;

4 points: 8–10 options selected;

Item I3:

0 points: “I do not think LLMs will have an impact”;

1 point: 1–2 options selected;

2 points: 3–4 options selected;

3 points: 5–6 options selected;

4 points: 7–9 options selected;

The final ATRAI-LLM questionnaire score is calculated as the sum of points from all scored items (T1, T3, T5, I1, I2, I3, H1, H2, and H3). The maximum possible total score is 36, and the minimum is 0.

## Appendix C. An Example of Questionnaire Completion with Total Score Calculation

1. P1 State your job title

∨	Physician (clinical specialist)
○	Head of department
○	Other

2. P2 Specialty

Urologist

3. P3 State the type of medical facility you work at

∨	Outpatient department
○	In-patient department
○	Day-case unit

4. P4 What is the age of the patients you work with?

○	<18 years old
∨	≥18 years old
○	I work with patients of all ages

5. P5 State your total years of professional experience (excluding residency)

○	Less than 1 year
∨	More than 1 year

If “More than 1 year,” specify the number of years

30

6. P6 Do you take part in research related to your medical specialty?

∨	Yes
○	No

7. P7 Do you use large language models (Gemini, ChatGPT, DeepSeek, etc.) in your clinical practice?

○	Yes
∨	No
○	No, but I would like to

8. F1 Do you use the question-answering LLM-based chatbot integrated into Health information system (hereinafter, LLM) in your clinical practice? (one answer)

	Answer
○	Yes, regularly, for various tasks (e.g., searching for drug information, interpreting test results, etc.)
○	Yes, regularly, but only for a specific clinical task (e.g., only for test–result interpretation or only for drug-information search)
○	Yes, but irregularly
∨	Not yet, but I plan to
○	No, and I do not plan to

9. T1 For which of the following topics would you trust the informational output from LLM?

∨	Regulatory documents, health information system functions, templates, sick-leave documentation
∨	Medications (INN, dosage forms, packaging, indications, dosing regimens, adverse reactions, contraindications, precautions, overdose, drug interactions)
∨	ICD-10 codes, diagnosis formulation, classifications, scoring systems, calculations
◻	Etiology, pathogenesis, epidemiology, clinical presentation, treatment of diseases
◻	General medical advice (diet, lifestyle, physical activity, etc.), vaccination, preparation for diagnostic tests
◻	Parameters, reference ranges, interpretation of diagnostic methods (physical examination, laboratory, and imaging data)
◻	I would not trust information on any topic
*Score for the item: 3.*	

10. T2 If LLM’s response raises doubts, would you cross-check the information? (one answer)

	Answer
∨	Yes
○	Mostly yes
○	Difficult to answer
○	Mostly no
○	No
*Score for the item: not for scoring, qualitative probe.*	

11. T3 Would you like LLMs for physicians to be widely implemented and actively used in clinical practice? (one answer)

	Answer
○	Yes
○	Mostly yes
∨	Difficult to answer
○	Mostly no
○	No
*Score for the item: 2.*	

12. T4 Which way of interacting with LLM would be preferable for you? (one answer)

	Answer
○	LLM provides both EHR-derived and reference information
○	LLM provides only reference information
○	LLM provides only EHR-derived information
∨	LLM provides EHR-derived and/or reference information with mandatory of source citations
○	I would prefer not to use an LLM within the health information system (e.g., UMIAS)
*Score for the item: not for scoring, qualitative probe.*	

13. T5 Do you trust the information provided by the LLM when it retrieves data from the patient’s EHR? (one answer)

○	Answer
○	Yes
○	Mostly yes
∨	Difficult to answer
○	Mostly no
○	No
*Score for the item: 2.*	

14. I1 In your opinion, which of the listed functions of LLM would be most useful and widely used in physicians’ work? (select all that applies)

∨	Summarizing the patient’s EHR for rapid review
∨	Providing reference information on regulatory documents, health information system functionality, templates, and sick-leave documentation
∨	Providing drug-related reference information
∨	Providing reference information on ICD-10 codes, diagnosis formulation, classifications, scoring systems, and calculations
∨	Providing reference information on parameters, reference ranges, and interpretation of diagnostic methods
◻	Providing reference information on etiology, pathogenesis, epidemiology, clinical presentation, and treatment of diseases
◻	Providing general medical advice (diet, lifestyle, physical activity, etc.), vaccination, and preparation for diagnostic tests
∨	Pre-populating medical documentation
◻	Assisting with communication with colleagues and patients
◻	I do not consider LLM useful
◻	Other (specify)
*Score for the item: 3.*	

15. I2 In your opinion, who should pay for access to LLM in healthcare? (one answer)

	Answer
∨	Public healthcare system (compulsory health insurance)
○	Patient’s insurance provider (voluntary health insurance)
○	The medical organization implementing LLM
○	The physician using LLM
○	LLM developer company
○	I do not anticipate widespread use of LLMs
○	Other (specify)
*Score for the item: 2.*	

16. I3 Which components of your daily professional activities do you expect to change with the use of LLMs in the next 1–2 years? (select all that applies)

∨	Communication with patients
◻	Communication with colleagues
∨	Searching medical information resources
∨	Completing medical documentation
∨	Clinical decision-making (patient management)
∨	Technical aspects of working with the health information system
∨	Interpretation of diagnostic results
∨	Analysis of patients’ EHRs
◻	I do not think LLMs will have an impact
◻	Other (specify)
*Score for the item: 4.*	

17. H1 In your opinion, will the widespread use of LLMs affect the prestige of the profession in the next five years? (one answer)

	Answer
○	Will not affect/difficult to answer
∨	Perhaps, the prestige will decrease slightly
○	Yes, the prestige will decrease substantially
○	Perhaps, the prestige will increase slightly
○	Yes, the prestige will increase substantially
*Score for the item: 1.*	

18. H2 In your opinion, will the widespread use of LLMs affect physicians’ workload? (one answer)

	Answer
○	The workload will decrease substantially
○	The workload will decrease somewhat
∨	Will not affect/difficult to answer
○	The workload will increase somewhat
○	The workload will increase substantially
*Score for the item: 2.*	

19. H3 In your opinion, will the widespread use of LLMs affect physicians’ salary over the next five years? (one answer)

	Answer
∨	Will not affect/difficult to answer
○	The salary will decrease somewhat
○	The salary will decrease substantially
○	The salary will increase somewhat
○	The salary will increase substantially
*Score for the item: 2.*	

*Total score: 22.*
